# (Acetyl­acetonato-κ^2^ *O*,*O*′)(2-bromo-4-chloro-6-{[2-(dimethyl­amino)­ethyl­imino]­meth­yl}phenolato-κ^3^ *N*,*N*′,*O*)oxidovanadium(IV)

**DOI:** 10.1107/S1600536811015406

**Published:** 2011-04-29

**Authors:** Fu-Ming Wang

**Affiliations:** a Department of Chemistry, Dezhou University, Dezhou Shandong 253023, People’s Republic of China

## Abstract

The V^IV^ atom in the title complex, [V(C_11_H_13_BrClN_2_O)(C_5_H_7_O_2_)O], is six-coordinated by one phenolate O, one imino N and one amino N atom of the tridentate anionic Schiff base ligand, by one oxide O atom, and by two O atoms of an acetyl­acetonate anion, forming a distorted *cis*-VN_2_O_4_ octa­hedral coordination geometry. The deviation of the V atom from the plane defined by the three donor atoms of the Schiff base ligand and one O atom of the acetyl­acetone ligand towards the oxide O atom is 0.256 (2) Å.

## Related literature

For background to oxidovanadium complexes, see: Hiromura *et al.* (2007 ▶); Seena *et al.* (2008 ▶); Rosenthal *et al.* (2008 ▶); Kurup *et al.* (2010 ▶). For similar oxidovanadium complexes with Schiff bases, see: Li *et al.* (1988 ▶); Cornman *et al.* (1992 ▶); Smith *et al.* (2000 ▶); Sarkar & Pal (2006 ▶).
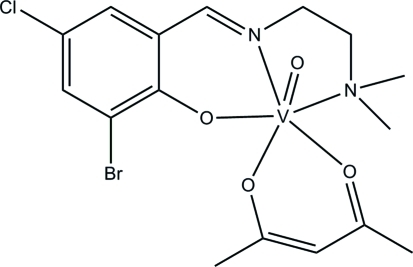


## Experimental

### 

#### Crystal data

[V(C_11_H_13_BrClN_2_O)(C_5_H_7_O_2_)O]
*M* *_r_* = 470.64
Orthorhombic, 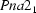

*a* = 20.351 (2) Å
*b* = 12.749 (1) Å
*c* = 7.410 (2) Å
*V* = 1922.6 (6) Å^3^
*Z* = 4
Mo *K*α radiation
μ = 2.76 mm^−1^
*T* = 298 K
0.37 × 0.33 × 0.32 mm

#### Data collection

Bruker SMART CCD diffractometer
Absorption correction: multi-scan (*SADABS*; Sheldrick, 1996 ▶) *T*_min_ = 0.429, *T*_max_ = 0.473
7060 measured reflections
3863 independent reflections
2284 reflections with *I* > 2σ(*I*)
*R*_int_ = 0.052

#### Refinement

*R*[*F* ^2^ > 2σ(*F* ^2^)] = 0.047
*wR*(*F* ^2^) = 0.100
*S* = 0.93
3863 reflections
230 parameters
1 restraint
H-atom parameters constrained
Δρ_max_ = 0.32 e Å^−3^
Δρ_min_ = −0.39 e Å^−3^
Absolute structure: Flack (1983 ▶), 1475 Friedel pairs
Flack parameter: 0.028 (14)

### 

Data collection: *SMART* (Bruker, 1998 ▶); cell refinement: *SAINT* (Bruker, 1998 ▶); data reduction: *SAINT*; program(s) used to solve structure: *SHELXS97* (Sheldrick, 2008 ▶); program(s) used to refine structure: *SHELXL97* (Sheldrick, 2008 ▶); molecular graphics: *SHELXTL* (Sheldrick, 2008 ▶); software used to prepare material for publication: *SHELXTL*.

## Supplementary Material

Crystal structure: contains datablocks global, I. DOI: 10.1107/S1600536811015406/hb5858sup1.cif

Structure factors: contains datablocks I. DOI: 10.1107/S1600536811015406/hb5858Isup2.hkl

Additional supplementary materials:  crystallographic information; 3D view; checkCIF report

## Figures and Tables

**Table:** 

V1—O4	1.598 (4)
V1—O1	1.952 (4)
V1—O3	1.988 (4)
V1—N1	2.078 (5)
V1—O2	2.168 (4)
V1—N2	2.222 (5)

**Table:** 

O4—V1—O1	100.0 (2)
O4—V1—O3	98.39 (19)
O1—V1—O3	88.92 (17)
O4—V1—N1	99.13 (19)
O1—V1—N1	88.30 (18)
O3—V1—N1	162.47 (18)
O4—V1—O2	173.0 (2)
O1—V1—O2	86.82 (17)
O3—V1—O2	82.86 (15)
N1—V1—O2	79.70 (17)
O4—V1—N2	91.3 (2)
O1—V1—N2	165.2 (2)
O3—V1—N2	98.79 (17)
N1—V1—N2	80.5 (2)
O2—V1—N2	81.70 (17)

**Fig. 1.**
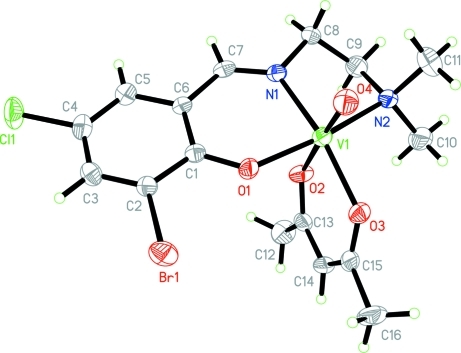
The molecular structure of the title compound, showing displacement ellipsoids drawn at the 30% probability level.

## supplementary crystallographic information

### Comment

Oxovanadium complexes have received much attention due to their structures and biological properties (Hiromura *et al.*, 2007; Seena *et al.*, 2008; Rosenthal *et al.*, 2008; Kurup *et al.*, 2010). In this paper, the title new oxovanadium(IV) complex, (I), with a Schiff base ligand is reported.

The V^IV^ atom in the title complex, Fig. 1, is six-coordinated by one phenolic O, one imino N, and one amino N atoms of the Schiff base ligand, by one oxo O atom, and by two O atoms of an acetylacetone ligand, forming a distorted octahedral geometry. The deviation of the V atom from the plane defined by the three donor atoms of the Schiff base ligand and one O atom of the acetylacetone ligand towards the oxo O atom is 0.256 (2) Å. The coordinate bond lengths and angles (Table 1) are comparable with those observed in similar oxovanadium(IV) complexes with Schiff bases and acetylacetone ligands (Li *et al.*, 1988; Cornman *et al.*, 1992; Smith *et al.*, 2000; Sarkar & Pal, 2006).

### Experimental

3-Bromo-5-chlorosalicylaldehyde (1 mmol, 0.23 g), *N,N*-dimethylethane-1,2-diamine (1 mmol, 0.09 g), and VO(acac)_2_ (1 mmol, 0.26 g) were mixed in methanol (30 ml). The mixture was boiled under reflux for 2 h, then cooled to room temperature. Green blocks of (I) were formed after slow evaporation of the solution in air for a few days.

### Refinement

Hydrogen atoms were placed in geometrically idealized positions and constrained to ride on their parent atoms, with C—H distances of 0.93–0.97 Å, and with *U*_iso_(H) set at 1.2*U*_eq_(C) and 1.5*U*_eq_(C_methyl_).

### Crystal data

**Table:**

[V(C_11_H_13_BrClN_2_O)(C_5_H_7_O_2_)O]	*D*_x_ = 1.626 Mg m^−^^3^
*M**_r_* = 470.64	Mo *K*α radiation, λ = 0.71073 Å
Orthorhombic, *P**n**a*2_1_	Cell parameters from 1129 reflections
*a* = 20.351 (2) Å	θ = 2.5–24.5°
*b* = 12.749 (1) Å	µ = 2.76 mm^−^^1^
*c* = 7.410 (2) Å	*T* = 298 K
*V* = 1922.6 (6) Å^3^	Block, green
*Z* = 4	0.37 × 0.33 × 0.32 mm
*F*(000) = 948	

### Data collection

**Table:**

Bruker SMART CCD diffractometer	3863 independent reflections
Radiation source: fine-focus sealed tube	2284 reflections with *I* > 2σ(*I*)
graphite	*R*_int_ = 0.052
ω scans	θ_max_ = 27.5°, θ_min_ = 2.6°
Absorption correction: multi-scan (*SADABS*; Sheldrick, 1996)	*h* = −15→26
*T*_min_ = 0.429, *T*_max_ = 0.473	*k* = −10→16
7060 measured reflections	*l* = −9→9

### Refinement

**Table:**

Refinement on *F*^2^	Secondary atom site location: difference Fourier map
Least-squares matrix: full	Hydrogen site location: inferred from neighbouring sites
*R*[*F*^2^ > 2σ(*F*^2^)] = 0.047	H-atom parameters constrained
*wR*(*F*^2^) = 0.100	*w* = 1/[σ^2^(*F*_o_^2^)]
*S* = 0.93	(Δ/σ)_max_ < 0.001
3863 reflections	Δρ_max_ = 0.32 e Å^−^^3^
230 parameters	Δρ_min_ = −0.39 e Å^−^^3^
1 restraint	Absolute structure: Flack (1983), 1475 Friedel pairs
Primary atom site location: structure-invariant direct methods	Flack parameter: 0.028 (14)

### Special details

**Table:**

Geometry. All esds (except the esd in the dihedral angle between two l.s. planes) are estimated using the full covariance matrix. The cell esds are taken into account individually in the estimation of esds in distances, angles and torsion angles; correlations between esds in cell parameters are only used when they are defined by crystal symmetry. An approximate (isotropic) treatment of cell esds is used for estimating esds involving l.s. planes.
Refinement. Refinement of F^2^ against ALL reflections. The weighted R-factor wR and goodness of fit S are based on F^2^, conventional R-factors R are based on F, with F set to zero for negative F^2^. The threshold expression of F^2^ > 2sigma(F^2^) is used only for calculating R-factors(gt) etc. and is not relevant to the choice of reflections for refinement. R-factors based on F^2^ are statistically about twice as large as those based on F, and R- factors based on ALL data will be even larger.

### Fractional atomic coordinates and isotropic or equivalent isotropic displacement parameters (Å^2^)

**Table:**

	*x*	*y*	*z*	*U*_iso_*/*U*_eq_	
V1	0.63268 (4)	−0.04041 (7)	0.00361 (16)	0.0381 (3)	
Br1	0.64969 (3)	0.21353 (5)	−0.47589 (10)	0.0583 (2)	
Cl1	0.51964 (10)	0.50695 (15)	−0.0592 (3)	0.0756 (6)	
N1	0.5724 (2)	0.0368 (4)	0.1869 (7)	0.0380 (12)	
N2	0.6403 (2)	−0.1505 (4)	0.2353 (7)	0.0439 (14)	
O1	0.62782 (18)	0.0834 (3)	−0.1510 (6)	0.0457 (11)	
O2	0.70569 (18)	0.0442 (3)	0.1592 (5)	0.0476 (11)	
O3	0.71095 (18)	−0.0874 (3)	−0.1350 (5)	0.0431 (10)	
O4	0.57889 (17)	−0.1130 (3)	−0.0917 (6)	0.0538 (12)	
C1	0.6018 (2)	0.1765 (5)	−0.1231 (8)	0.0343 (14)	
C2	0.6071 (3)	0.2535 (5)	−0.2607 (8)	0.0376 (15)	
C3	0.5833 (3)	0.3523 (6)	−0.2406 (9)	0.0450 (17)	
H3	0.5897	0.4019	−0.3310	0.054*	
C4	0.5496 (3)	0.3788 (5)	−0.0854 (10)	0.0501 (17)	
C5	0.5398 (2)	0.3065 (5)	0.0468 (9)	0.0426 (15)	
H5	0.5161	0.3248	0.1495	0.051*	
C6	0.5652 (2)	0.2037 (4)	0.0296 (11)	0.0375 (12)	
C7	0.5528 (3)	0.1311 (5)	0.1766 (8)	0.0402 (15)	
H7	0.5280	0.1562	0.2728	0.048*	
C8	0.5571 (3)	−0.0253 (5)	0.3450 (10)	0.0534 (19)	
H8A	0.5201	−0.0709	0.3202	0.064*	
H8B	0.5456	0.0203	0.4449	0.064*	
C9	0.6162 (3)	−0.0896 (6)	0.3927 (9)	0.060 (2)	
H9A	0.6509	−0.0436	0.4353	0.072*	
H9B	0.6051	−0.1374	0.4898	0.072*	
C10	0.7074 (3)	−0.1882 (6)	0.2724 (10)	0.072 (2)	
H10A	0.7069	−0.2319	0.3779	0.108*	
H10B	0.7359	−0.1294	0.2923	0.108*	
H10C	0.7230	−0.2280	0.1711	0.108*	
C11	0.5987 (3)	−0.2457 (5)	0.2077 (13)	0.076 (2)	
H11A	0.5551	−0.2247	0.1734	0.113*	
H11B	0.5967	−0.2852	0.3179	0.113*	
H11C	0.6174	−0.2883	0.1142	0.113*	
C12	0.8031 (3)	0.1328 (6)	0.2472 (10)	0.064 (2)	
H12A	0.8117	0.0954	0.3572	0.097*	
H12B	0.7777	0.1944	0.2733	0.097*	
H12C	0.8439	0.1527	0.1923	0.097*	
C13	0.7652 (3)	0.0633 (5)	0.1198 (9)	0.0422 (16)	
C14	0.7966 (3)	0.0228 (5)	−0.0327 (9)	0.0459 (18)	
H14	0.8394	0.0447	−0.0556	0.055*	
C15	0.7685 (3)	−0.0473 (5)	−0.1516 (9)	0.0459 (17)	
C16	0.8073 (3)	−0.0823 (6)	−0.3127 (10)	0.069 (2)	
H16A	0.7848	−0.0620	−0.4209	0.104*	
H16B	0.8121	−0.1572	−0.3101	0.104*	
H16C	0.8500	−0.0501	−0.3101	0.104*	

### Atomic displacement parameters (Å^2^)

**Table:**

	*U*^11^	*U*^22^	*U*^33^	*U*^12^	*U*^13^	*U*^23^
V1	0.0346 (4)	0.0410 (6)	0.0388 (6)	0.0030 (4)	0.0017 (5)	−0.0003 (6)
Br1	0.0701 (4)	0.0680 (5)	0.0367 (3)	−0.0054 (3)	0.0078 (4)	−0.0003 (5)
Cl1	0.1159 (14)	0.0445 (11)	0.0665 (12)	0.0281 (11)	0.0116 (11)	0.0049 (10)
N1	0.031 (3)	0.041 (3)	0.042 (3)	−0.001 (2)	0.006 (2)	−0.001 (3)
N2	0.040 (3)	0.043 (4)	0.049 (3)	0.004 (3)	0.010 (2)	0.010 (3)
O1	0.057 (2)	0.043 (3)	0.038 (2)	0.014 (2)	0.018 (2)	0.004 (2)
O2	0.038 (2)	0.061 (3)	0.044 (3)	−0.005 (2)	0.0084 (19)	−0.012 (2)
O3	0.043 (2)	0.043 (3)	0.043 (3)	0.003 (2)	0.004 (2)	−0.006 (2)
O4	0.042 (2)	0.053 (3)	0.066 (3)	−0.002 (2)	−0.012 (2)	−0.005 (2)
C1	0.027 (3)	0.040 (4)	0.036 (4)	−0.002 (3)	−0.005 (2)	−0.001 (3)
C2	0.036 (3)	0.042 (4)	0.035 (4)	0.000 (3)	−0.002 (3)	0.002 (3)
C3	0.052 (4)	0.045 (5)	0.038 (4)	−0.004 (3)	−0.007 (3)	0.005 (3)
C4	0.053 (4)	0.042 (4)	0.056 (5)	0.005 (3)	−0.011 (3)	0.000 (4)
C5	0.047 (3)	0.049 (4)	0.033 (4)	0.004 (3)	−0.005 (3)	−0.003 (4)
C6	0.032 (2)	0.040 (3)	0.041 (3)	0.003 (2)	0.006 (4)	−0.001 (4)
C7	0.032 (3)	0.052 (5)	0.037 (4)	0.003 (3)	0.008 (3)	−0.005 (3)
C8	0.052 (4)	0.045 (4)	0.063 (5)	0.006 (3)	0.027 (3)	0.017 (4)
C9	0.068 (4)	0.062 (5)	0.051 (5)	0.012 (4)	0.020 (4)	0.021 (4)
C10	0.053 (4)	0.076 (6)	0.087 (6)	0.019 (4)	0.008 (4)	0.029 (5)
C11	0.089 (5)	0.054 (5)	0.084 (6)	−0.015 (4)	−0.001 (5)	0.006 (5)
C12	0.055 (4)	0.065 (6)	0.073 (6)	−0.008 (4)	−0.011 (4)	−0.017 (5)
C13	0.037 (4)	0.041 (4)	0.049 (4)	0.000 (3)	0.001 (3)	0.013 (3)
C14	0.032 (3)	0.050 (4)	0.056 (5)	−0.001 (3)	0.009 (3)	0.006 (3)
C15	0.051 (4)	0.045 (5)	0.042 (4)	0.017 (4)	0.007 (3)	0.019 (4)
C16	0.069 (5)	0.090 (6)	0.049 (4)	0.010 (4)	0.030 (4)	−0.001 (5)

### Geometric parameters (Å, °)

**Table:**

V1—O4	1.598 (4)	C6—C7	1.452 (9)
V1—O1	1.952 (4)	C7—H7	0.9300
V1—O3	1.988 (4)	C8—C9	1.497 (8)
V1—N1	2.078 (5)	C8—H8A	0.9700
V1—O2	2.168 (4)	C8—H8B	0.9700
V1—N2	2.222 (5)	C9—H9A	0.9700
Br1—C2	1.885 (6)	C9—H9B	0.9700
Cl1—C4	1.755 (7)	C10—H10A	0.9600
N1—C7	1.268 (7)	C10—H10B	0.9600
N1—C8	1.448 (8)	C10—H10C	0.9600
N2—C10	1.473 (7)	C11—H11A	0.9600
N2—C9	1.484 (8)	C11—H11B	0.9600
N2—C11	1.494 (7)	C11—H11C	0.9600
O1—C1	1.316 (6)	C12—C13	1.507 (8)
O2—C13	1.269 (6)	C12—H12A	0.9600
O3—C15	1.285 (7)	C12—H12B	0.9600
C1—C6	1.399 (9)	C12—H12C	0.9600
C1—C2	1.420 (8)	C13—C14	1.397 (8)
C2—C3	1.358 (8)	C14—C15	1.379 (8)
C3—C4	1.381 (8)	C14—H14	0.9300
C3—H3	0.9300	C15—C16	1.499 (9)
C4—C5	1.360 (8)	C16—H16A	0.9600
C5—C6	1.414 (7)	C16—H16B	0.9600
C5—H5	0.9300	C16—H16C	0.9600
			
O4—V1—O1	100.0 (2)	C6—C7—H7	116.7
O4—V1—O3	98.39 (19)	N1—C8—C9	108.6 (5)
O1—V1—O3	88.92 (17)	N1—C8—H8A	110.0
O4—V1—N1	99.13 (19)	C9—C8—H8A	110.0
O1—V1—N1	88.30 (18)	N1—C8—H8B	110.0
O3—V1—N1	162.47 (18)	C9—C8—H8B	110.0
O4—V1—O2	173.0 (2)	H8A—C8—H8B	108.4
O1—V1—O2	86.82 (17)	N2—C9—C8	111.4 (6)
O3—V1—O2	82.86 (15)	N2—C9—H9A	109.3
N1—V1—O2	79.70 (17)	C8—C9—H9A	109.3
O4—V1—N2	91.3 (2)	N2—C9—H9B	109.3
O1—V1—N2	165.2 (2)	C8—C9—H9B	109.3
O3—V1—N2	98.79 (17)	H9A—C9—H9B	108.0
N1—V1—N2	80.5 (2)	N2—C10—H10A	109.5
O2—V1—N2	81.70 (17)	N2—C10—H10B	109.5
C7—N1—C8	120.0 (5)	H10A—C10—H10B	109.5
C7—N1—V1	126.5 (4)	N2—C10—H10C	109.5
C8—N1—V1	113.3 (4)	H10A—C10—H10C	109.5
C10—N2—C9	109.3 (5)	H10B—C10—H10C	109.5
C10—N2—C11	106.6 (5)	N2—C11—H11A	109.5
C9—N2—C11	110.2 (5)	N2—C11—H11B	109.5
C10—N2—V1	114.5 (4)	H11A—C11—H11B	109.5
C9—N2—V1	104.7 (3)	N2—C11—H11C	109.5
C11—N2—V1	111.6 (4)	H11A—C11—H11C	109.5
C1—O1—V1	131.1 (4)	H11B—C11—H11C	109.5
C13—O2—V1	128.8 (4)	C13—C12—H12A	109.5
C15—O3—V1	131.3 (4)	C13—C12—H12B	109.5
O1—C1—C6	124.5 (5)	H12A—C12—H12B	109.5
O1—C1—C2	118.7 (5)	C13—C12—H12C	109.5
C6—C1—C2	116.7 (6)	H12A—C12—H12C	109.5
C3—C2—C1	122.4 (6)	H12B—C12—H12C	109.5
C3—C2—Br1	120.5 (5)	O2—C13—C14	123.5 (6)
C1—C2—Br1	117.1 (5)	O2—C13—C12	117.1 (6)
C2—C3—C4	119.7 (6)	C14—C13—C12	119.3 (5)
C2—C3—H3	120.1	C15—C14—C13	124.5 (5)
C4—C3—H3	120.1	C15—C14—H14	117.7
C5—C4—C3	120.5 (6)	C13—C14—H14	117.7
C5—C4—Cl1	120.0 (5)	O3—C15—C14	125.1 (6)
C3—C4—Cl1	119.5 (6)	O3—C15—C16	116.0 (6)
C4—C5—C6	120.6 (6)	C14—C15—C16	118.9 (6)
C4—C5—H5	119.7	C15—C16—H16A	109.5
C6—C5—H5	119.7	C15—C16—H16B	109.5
C1—C6—C5	119.9 (6)	H16A—C16—H16B	109.5
C1—C6—C7	122.7 (5)	C15—C16—H16C	109.5
C5—C6—C7	117.4 (6)	H16A—C16—H16C	109.5
N1—C7—C6	126.5 (6)	H16B—C16—H16C	109.5
N1—C7—H7	116.7		

## References

[bb1] Bruker (1998). *SMART* and *SAINT* Bruker AXS Inc., Madison, Wisconsin, USA.

[bb2] Cornman, C. R., Kampf, J., Lah, M. S. & Pecoraro, V. L. (1992). *Inorg. Chem.* **31**, 2035–2043.

[bb3] Flack, H. D. (1983). *Acta Cryst.* A**39**, 876–881.

[bb4] Hiromura, M., Nakayama, A., Adachi, Y., Doi, M. & Sakurai, H. (2007). *J. Biol. Inorg. Chem.* **12**, 1275–1287. 10.1007/s00775-007-0295-x 17805585

[bb5] Kurup, M. R. P., Seena, E. B. & Kuriakose, M. (2010). *Struct. Chem.* **21**, 599–605.

[bb6] Li, X., Lah, M. S. & Pecoraro, V. L. (1988). *Inorg. Chem.* **27**, 4657–4664.

[bb7] Rosenthal, E. C. E., Cui, H. L. & Hummert, M. (2008). *Inorg. Chem. Commun.* **11**, 918–920.

[bb8] Sarkar, A. & Pal, S. (2006). *Polyhedron*, **25**, 1689–1694.

[bb9] Seena, E. B., Mathew, N., Kuriakose, M. & Kurup, M. R. P. (2008). *Polyhedron*, **27**, 1455–1462.

[bb10] Sheldrick, G. M. (1996). *SADABS* University of Göttingen, Germany.

[bb11] Sheldrick, G. M. (2008). *Acta Cryst.* A**64**, 112–122. 10.1107/S0108767307043930 18156677

[bb12] Smith, T. S., Root, C. A., Kampf, J. W., Rasmussen, P. G. & Pecoraro, V. L. (2000). *J. Am. Chem. Soc.* **122**, 767–775.

